# Study protocol of a multicenter randomized controlled trial of mindfulness training to reduce burnout and promote quality of life in police officers: the POLICE study

**DOI:** 10.1186/s12888-018-1726-7

**Published:** 2018-05-25

**Authors:** Marcelo Trombka, Marcelo Demarzo, Daniel Campos Bacas, Sonia Beira Antonio, Karen Cicuto, Vera Salvo, Felipe Cesar Almeida Claudino, Letícia Ribeiro, Michael Christopher, Javier Garcia-Campayo, Neusa Sica Rocha

**Affiliations:** 1 Hospital de Clínicas de Porto Alegre, 2350, Ramiro Barcelos Street, Porto Alegre, Brazil; 2 Mente Aberta - Brazilian Center for Mindfulness and Health Promotion - Universidade Federal de São Paulo, 545, Padre José Maria Avenue, São Paulo, Brazil; 3 Universitat Jaume I, Vicent Sos Baynat Avenue, Castelló de la Plana, Spain; 4 Pacific University, 2043 College Way, Forest Grove, Oregon USA; 5 Miguel Servet Hospital, 1-3, Paseo Isabel la Católica Street, Zaragoza, Spain

**Keywords:** Mindfulness, Police, Quality of life, Burnout, Stress

## Abstract

**Background:**

Police officers experience a high degree of chronic stress. Policing ranks among the highest professions in terms of disease and accident rates. Mental health is particularly impacted, evidenced by elevated rates of burnout, anxiety and depression, and poorer quality of life than the general public. Mindfulness training has been shown to reduce stress, anxiety, burnout and promote quality of life in a variety of settings, although its efficacy in this context has yet to be systematically evaluated. Therefore, this trial will investigate the efficacy of a mindfulness-based intervention versus a waitlist control in improving quality of life and reducing negative mental health symptoms in police officers.

**Methods:**

This multicenter randomized controlled trial has three assessment points: baseline, post-intervention, and six-month follow-up. Active police officers (*n* = 160) will be randomized to Mindfulness-Based Health Promotion (MBHP) or waitlist control group at two Brazilian major cities: Porto Alegre and São Paulo. The primary outcomes are burnout symptoms and quality of life. Consistent with the MBHP conceptual model, assessed secondary outcomes include perceived stress, anxiety and depression symptoms, and the potential mechanisms of resilience, mindfulness, decentering, self-compassion, spirituality, and religiosity.

**Discussion:**

Findings from this study will inform and guide future research, practice, and policy regarding police offer health and quality of life in Brazil and globally.

**Trial registration:**

ClinicalTrials.gov NCT03114605. Retrospectively registered on March 21, 2017.

## Background

Police officers are routinely exposed to dangerous and unpredictable situations [1]. Exposure to acute and chronic stress is reflected in elevated officer rates of burnout [2, 3], depression [4], suicide [5–7], and anxiety [8]. In addition to higher rates of mental health concerns, police officers have an increased risk of sudden cardiac death, diabetes, obesity and metabolic syndrome [9, 10]. It is no surprise to find that this occupation ranks highest on diseases and accidents rates among all professions [11, 12]. Police quality of life is generally poor [13], including among Brazilian military police [14, 15] and recruits [16]. Brazil has the 7th highest crime index in the world, and Porto Alegre and São Paulo—where participants will be recruited for this study—have the 11th and 14th highest crime index of cities in the world, respectively [17, 18].

Police stress and related mental and physical health consequences can negatively impact work and personal life. Stress-impaired officers are more likely to make serious errors, and exhibit other adverse work outcomes including uncontrolled anger toward suspects, excessive use of force, and absenteeism [19, 20]. The effects of work-related stress also extends to personal life, contributing to disruptive family relationships [21]. It is estimated that approximately one third of police officers meet criteria for burnout syndrome (emotional exhaustion, despersonalization, and low personal accomplishment) [22]. The emotional, physical, economic and social damage of stress in police indicates an urgent call for effective preventive programs to reduce the negative impact of stress and burnout, and to enhance quality of life and well-being.

Mindfulness is an innate meta-awareness capacity to attend to present-moment experience while avoiding entanglement in cognitive biases. It can be enhanced by simple meditative attentional practices, as well as through structured training programs such as mindfulness-based interventions (MBIs). MBIs are generally group-based weekly classes consisting primarily of meditation practices, such as the body scan, mindful movement, sitting and walking meditation. By repeatedly bringing attention back to the current experience, participants gradually learn to disengage from “automatic pilot”, and directly experience emotions and bodily sensations in the present moment. In MBIs, participants learn the ability to step back from ruminating about the past or worrying about the future and simply allow experiences to unfold [23, 24].

Mindfulness training may regulate how the individual appraises stress, and increase secondary appraisals of approach-oriented coping resources, thus reducing stress reactivity. Recent meta-analyses support this theory, indicating MBIs improve common health and risk factors, including stress [25, 26], depression and suicidal ideation [26, 27], anxiety and burnout [28, 29], and increases psychological resilience [30, 31]. MBIs have been shown to be feasible and to lead to improved health outcomes among several high-stress populations, such as military personnel [32, 33], physicians [34, 35], firefighters [36], and inner-city teachers [37].

MBI studies among police officers are limited; however, a pre-post study suggests that mindfulness training can reduce stress and burnout symptoms [38], and findings from a recent RCT indicate that relative to waitlist control, MBI participants experienced greater reductions in salivary cortisol (AUCi), self-reported aggression, burnout, alcohol use, sleep disturbance, and increases in psychological flexibility and non-reactivity at post-training [39]. Despite these promising findings and a global increase among law enforcement agencies integrating mindfulness into trainings, the impact and benefits of MBIs have yet to be systematically evaluated in this context.

### Aims

The aim of the POLICE (imPact Of a mindfuLness-based Intervention on burnout and quality of life in poliCE officers) study is to evaluate the efficacy of a MBI, compared to a waitlist control group, in promoting quality of life and mental health in police officers. We also aim to investigate the feasibility and acceptability of an 8-week, 2-h long MBI on this particular setting.

Our main hypothesis is that mindfulness training will reduce burnout symptoms and improve quality of life. We expect individuals allocated to the MBI group will endorse improved quality of life and reduced burnout (primary outcomes), as well as reduced stress, anxiety, and depression symptoms (secondary outcomes). Potential mechanisms of change include mindfulness, self-compassion, spirituality, decentering, and resilience skills.

## Methods

### Study design

**Fig. 1:**
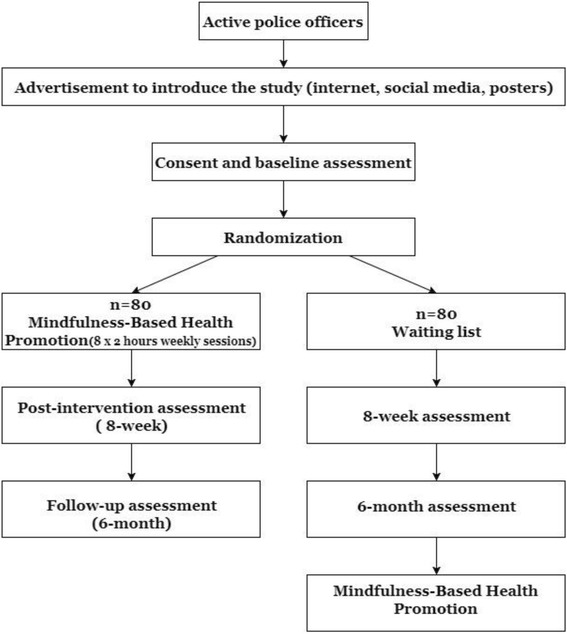
Study design

The POLICE study is a multicenter, parallel, two-armed, RCT with 3 assessment points (baseline, post-intervention, and six-month follow-up). Participants will be randomized to MBI or a waiting list control group. This protocol was prepared in accordance with SPIRIT 2013 statement [40] and was approved by the centers ethical committees. The study was registered under ClinicalTrials.gov NCT03114605 on 03/21/17 and will be conducted following the CONSORT guidelines [41]. Figure 1 shows the participant flow through the trial.

### Sample size and power calculations

Power calculations were conducted to determinate the required sample size for the primary outcomes using the statistical software G * Power 3.1.9.2. The minimum sample for each group was identified as 64 to detect a moderate effect size of 0.5 (Cohen’s *d*) with a power of .80 and an alpha of 0.05, based on a previous study [42]. Therefore, the RCT will enroll 160 participants (80 each group) and allow for up to 20% loss to follow-up, expecting a minimum effective sample size of 128 individuals.

### Participants and settings

Two types of police officers will be recruited for the POLICE study in two Brazilian metropolitan areas:

1. *Civil Police.* The study center in Porto Alegre will be located in the Police Hall of the State of Rio Grande do Sul. Civil Police main duties are to oversee public order and security, adopt measures necessary to avoid danger or injury to persons and public or private property, ensure the administration of criminal offenses, including execution of arrest warrants and requisitions requested by the judicial branch.
2. *Civil Guard.* The study center in São Paulo will be located in the headquarters of Unifesp Santo Amaro Campus. Civil Guard fundamental duties include the protection of goods, services and municipal facilities, as well as patrolling areas and preventive policing.

### Eligibility

To be admitted in the study, participants will meet the following inclusion criteria: (a) be active police officers; (b) 21 to 65 years old; (c) available to attend 8 sessions; (d) willing to voluntarily participate. Exclusion criteria include: (a) previous involvement in any mindfulness-based intervention or being practicing mindfulness regularly in the last 3 months, or one of the following diagnosis, as evaluated by a trained psychologist using the Mini International Neuropsychiatric Interview (MINI) [43]: (b) major depressive episode (current); (c) manic or hypomaniac episode (current); (d) psychotic syndrome (current or past); (e) substance use disorder (past 12 months, except tobacco); (f) risk of suicide.

### Recruitment, procedures and randomization

We will recruit 160 police officers from 2016 to 2018. Recruitment will be conducted using advertisements within the participating police organizations (Internet, social media, posters). Interested officers will contact the research team. After the first screening by phone or e-mail, potential participants will be scheduled for a face-to face interview, where they will be provided with detailed information about the study, fill the informed consent form and inclusion/exclusion criteria will be evaluated. Successful volunteers will be randomly assigned to the MBI or waitlist. Randomization will be implemented using sealed envelopes by an independent researcher who will be unaware of the characteristics of the study and will have no involvement in the trial or access to the study data. Participants allocated to the waitlist will not participate in any active intervention during this study period; however, they offered the MBI program after the 6-month follow-up. Each intervention and waitlist control group will be composed of around 20 participants. Due to the nature of the intervention, this will be a single-blind study, in which the outcomes assessment will be blind, but participants will be aware of their group assignment.

### Interventionists

Two interventionists will lead the MBI groups - one in Porto Alegre and one in São Paulo. Both have been certified by Mente Aberta - Brazilian Center for Mindfulness and Health Promotion to teach mindfulness courses. This rigorous training follows the British and Brazilian guidelines for good practices of mindfulness [44, 45]. An independent experienced mindfulness teacher will assess intervention fidelity using video recordings of the sessions.

### Intervention

The intervention in the POLICE study will be the Mindfulness-Based Health Promotion (MBHP) program [46]. MBHP is an 8-week MBI developed by our research group (see Table 1), based on the Mindfulness-Based Stress Reduction (MBSR) model created by Jon Kabat-Zinn and colleagues [47]. We adapted the MBSR model in order to suit the context and needs of primary health care and national and local health systems, developing a framework that supports the learning process to individuals from distinctive cultural and educational backgrounds. MBHP was designed to address human universal vulnerabilities, not focusing in any specific health condition. It has been applied over the last years by Mente Aberta - Brazilian Center for Mindfulness and Health Promotion [48], and by the University of Zaragoza, in Spain [49]. There is a strong emphasis on informal mindfulness practices, such as walking, eating, exercising, talking and doing housework. Concepts of radical acceptance [50], values clarification [51] and positive psychology [52] are also heavily emphasized. Compassion training techniques are also included in the program (equanimity in the condition of human suffering, receiving affection and showing affection to oneself), which can be particularly useful for persons high on guilt or self-criticism like police officers.

**Table 1:** Mindfulness-Based Health Promotion content

Session	Didactive teaching	Practices	Homework
1. What is mindfulness	-Presentation and aims-Stress and dispersion-What is and what isn’t mindfulness-Characteristics, attitudes and motivation-Introduction to practice diaries	-Eating a raisin-Simple mindfulness exercises (listening to sounds, areas of contact with your body, the feel of your feet touching the floor)-Body scan-9-dots exercise	-Body scan-Attention for routine activity-Eating one meal mindfully-Mindfulness diary
2. Mindfulness in breathing	-What to do with the body-What to do with the mind-Breathing-Information on posture-Preconceptions/fears/challenges in meditation	-Mindfulness in breathing-Body scan-Primary and secondary suffering	-Body scan-Mindfulness in breathing-Awareness of pleasant events (diary)-Attention for routine activity-Habit changing
3. Mindfulness in the body (part I)	-Importance of the body-How mindfulness works-Cognitive defusion	-Mindful walking-Mindfulness in breathing-Hello, thank you and goodbye	-Body scan-Mindfulness in breathing-Mindful walking-Awareness of unpleasant events (diary)-Attention for routine walking
4. Mindfulness in the body (part II)	-Doing mode/being mode-Reinforcing daily practice-Obstacles to the practice-Self -compassion	-Mindful movements -Mindfulness in breathing, sensations, sounds and thoughts-3-min breathing space-Mindful walking	-Body scan-Mindfulness in breathing-Mindful walking-3-min breathing space-Awareness of communication difficulties (diary)
5. Mindfulness, acceptance and values	-Importance of acceptance-Values and committed action-Meaning of life-Hedonic and eudaimonic well-being	-Mindful movements-Mindfulness of thoughts-3-min breathing space in doubles	-Practice of choice-3-min breathing space during stress
6. Silence		-Mindful movements-Body scan-Sitting meditation-Mindful walking	-Practice of choice-Mindfulness conversation
7. Compassion	-What is and what isn’t compassion-Biological basis-Ways of training compassion-Fear of compassion in western society	-Loving-kindness (for oneself and others)-Sitting meditation-Mindful movements	-Practice of choice- Loving-kindness (for oneself and others)-Attention for self-compassion in routine activity
8. Mindfulness for life	-Mindfulness in daily life-Recommendation for long-term practice	-Loving-kindness (for oneself and others)-Mindfulness poetry	-Daily life mindfulness incorporation

### Assessments and outcomes

The three assessment points—baseline, post-intervention and follow-up—will occur over the two weeks preceding the intervention, the two weeks after the intervention, and six months after the intervention, respectively (see Table 2). The baseline assessment also will include sociodemographic and clinical characteristics. Survey Monkey Software [53] will be used for data collection. During research visits the participants will enter responses directly into the electronic system. Whenever this may not be possible, the research team will provide sheets of paper and the answers will be uploaded by the team. All instruments have been translated to Portuguese with reliable psychometric properties [54–64].

**Table 2:** Assessments as a function of time points (according to 2013 SPIRIT figure guidelines)

Measure	Target	T0	T1	T2
		Baseline	Post-intervention (8 weeks)	Follow-up (6 months)
*MINI*	Psychiatry disorders	x		
*WHOQOL-BREF*	Quality of life	x	x	X
*MBI-GS*	Burnout symptoms	x	x	X
*PSQ*	Stress	x	x	X
*HADS*	Anxiety and depression symptoms	x	x	X
*CD-RISC-25*	Resilience	x	x	X
*MAAS*	Mindfulness trait	x	x	X
*EQ*	Decentering	x	x	X
*SCS*	Self-compassion	x	x	X
*GHQ-12*	Mental well-being	x	x	X
*BCSQ-12*	Burnout subtypes	x	x	X
*WHOQOL-SRPB-BREF*	Spirituality, religiosity and personal beliefs	x	x	X
*DUREL*	Religiosity	x	x	X

*MINI* Mini International Neuropsychiatric Interview, *WHOQOL-BREF* World Health Organization Quality of Life bref, *MBI-GS* Maslach Burnout Inventory - General Survey, *PSQ* Police Stress Questionnaire, *HADS* Hospital Anxiety and Depression Scale, *CD-RISC-25* Connor-Davidson-25 Scale of Resilience, *MAAS* Mindful Attention Awareness Scale, *EQ* Experience Questionnaire, *SCS* Self Compassion Scale, *GHQ-12* The 12-Item General Health Questionnaire, *BCSQ-12* Burnout Clinical Subtype Questionnaire, *WHOQOL-SRPB-BREF* World Health Organization Quality of Life Bref -Spirituality, Religiousness and Personal Beliefs, *DUREL* Duke University Religion Index

### Diagnostic interview

The Mini International Neuropsychiatric Interview (MINI) is a short, structured diagnostic interview that is used for the diagnosis of DSM-IV and ICD-10 psychiatric disorders. With an administration time of approximately 15 min, the MINI was designed to provide a short but accurate structured psychiatric interview for multicentric clinical trials and epidemiology studies [43].

### Primary outcomes measures

- *World Health Organization Quality of Life- BREF (WHOQOL-BREF):* the World Health Organization has developed a quality of life instrument, the WHOQOL, which captures many subjective aspects of quality of life. The WHOQOL-BREF is a WHOQOL shorter version, comprises 26 items that produces scores for four domains related to quality of life: physical health, psychological, social relationships and environment. It also includes one facet on overall quality of life and general health. Is one of the best known instruments that has been developed for cross-cultural comparisons of quality of life and is available in more than 40 languages [65]. Evidence consistently shows that officers exhibit low quality of life and its correlation with adverse mental health outcomes [4, 66, 67].
- *Maslach Burnout Inventory - General Survey (MBI-GS):* is considered one of the leading measures of burnout. Validated by extensive research, MBI-GS Surveys address three dimensions: Emotional Exhaustion measures feelings of being emotionally overextended and exhausted by one’s work; Depersonalization measures an unfeeling and impersonal response toward recipients of one’s service, care treatment, or instruction; Personal Accomplishment measures feelings of competence and successful achievement in one’s work. The MBI-GS is designed for use with occupational groups. The 3 dimensions are distributed in 16 items, whose answers are given on a 7-point Likert scale that varies from “never” to “every day” [68]. Nature and environment of police work contributes to high burnout rates which are associated with escalating symptoms of diminished health [22].

### Secondary outcomes measures

- *Police Stress Questionnaire (PSQ) -* stress will be measured using the PSQ, a 40-item psychometric questionnaire consisting of two subscales that measure organizational and operational stressors in the context of police work, using 7-point scales (Likert type) ranging from 1 (absence of stress) to 7 extreme stress). The PSQ was developed as an alternative to the general scales of stress at work in order to reflect the specifics stressors of police occupation [69].
- *Hospital Anxiety and Depression Scale (HADS)* - is a 14-item questionnaire with seven items measuring anxiety (HAD-A) and seven measuring depression (HAD-D). The ratings are totaled to obtain a score ranging from 0 to 21 for anxiety and depression, with higher scores indicating greater depression or anxiety. Scores between 8 and 10 represent possible cases of anxiety or depression, and scores that are ≥11 correspond to probable cases [70].
- *Connor-Davidson-25 Scale of Resilience (CD-RISC-25)*: it assesses the ability of individuals to cope and adapt physically, mentally, and spiritually to the circumstances imposed by life. CD-RISC-25 is composed of 25 items on a Likert scale (0–4), with larger scores reflecting greater resilience. Resilience may be viewed as a measure of stress coping ability and, as such, could be an important target of treatment in anxiety, depression, and stress reactions [71].
- *Mindful Attention Awareness Scale (MAAS)*: is a 15-item scale designed to assess a core characteristic of dispositional mindfulness, namely, open or receptive awareness of and attention to what is occurring in the present. The scale has strong psychometric properties and has been validated with college, community, and cancer patient samples [72].
- *Experience Questionnaire (EQ)*: this instrument was designed to measure the capacity to observe one’s thoughts and feelings as temporary and objective events of the mind, also known as “decentering”. Items from the scale are rated on a Likert scale ranging between 1 (never or very rarely true) and 5 (very often or always true), with higher scores indicating greater decentering [73].
- *Self Compassion Scale (SCS):* is composed by 26 items measuring six components of self-compassion: self-kindness, self-judgment, common humanity, isolation, mindfulness and over-identification.The items are rated on a five-point response scale ranging from 1 (almost never) to 5 (almost always) [74].
- *The 12-Item General Health Questionnaire (GHQ-12)*: is the most extensively used screening instrument for common mental disorders, in addition to being a more general measure of mental well-being. It consists of 12 items, whose answers are given on a 4-point Likert scale [75].
- *Burnout Clinical Subtype Questionnaire (BCSQ-12):* it aims to evaluate the most important clinical subtypes of burnout, comprising the dimensions of overload, lack of development and neglect. Consists of 12 items distributed in 3 dimensions, whose answers are given on a 7-point Likert scale ranging from “totally agree” to “totally disagree” [76].
- *WHOQOL-SRPB-BREF*: this scale measures the domains of spirituality, religiosity and personal beliefs in the quality of life from a cross-cultural perspective. Combines one item extracted from each of the eight SRPB facets in the WHOQOL-SRPB, with the 26 WHOQOL-BREF items, extracted from the WHOQOL-100, to construct the 34 item WHOQOL-SRPB-BREF [77].
- *Duke University Religion Index (DUREL)*: scale of religiousness developed by Duke University, 5 items are used to evaluate religious involvement. It captures the three dimensions of religiosity that most correlate with health outcomes: organizational religiosity, non-organizational religiosity, and intrinsic religiosity (or subjective religiosity) [78].

To explore the feasibility and adherence of MBHP among police officers, the frequency of attendance to the sessions will be assessed. At the post-intervention and the six-month follow-up evaluations we will address mindfulness practice parameters such as type, frequency and length of personal meditative practice through a questionnaire created by our research group [79].

### Statistical analysis

Intention-to-treat (ITT) and per protocol analyses (PPA) will be conducted following the CONSORT recommendations for reporting the results [41]. Baseline differences between groups on sociodemographic and clinical variables will be assessed using Chi-square tests for categorical variables and analysis of variance (ANOVA) for continuous data. A 2 (Group: MBI vs. WL) × 3 (Time: baseline, post, and 6-month follow-up) repeated measures ANOVA will be conducted to test the effects of the intervention for all primary and secondary outcomes. Effect sizes (Cohen’s *d*) and confidence intervals for within- and between-group comparisons will be performed for each study outcome. Missing data will be handled according to the most appropriate method depending on the reasons for missingness, following the authors’ recommendations and sensitivity analyses principles [80].

To study potential working mechanisms of mindfulness intervention, mediation analysis will be conducted using the Preacher and Hayes (2004) procedure [81]. The mediation effects of mindfulness, self-compassion, resilience, decentering and spirituality on burnout symptoms and quality of life will be evaluated.

### Ethical considerations

The study is registered at ClinicalTrials.gov as NCT03114605 and we have adopted the SPIRIT Statement recommendations for Clinical Trials [40]. Declaration of Helsinki protocols are being followed and patients will give written informed consent. The study was approved by HCPA and UNIFESP Research Ethics Committees under number 60406416.9.1001.5327.

### Trial status

At the time of this manuscript submission, recruitment for the POLICE study is ongoing.

## Discussion

To our knowledge, the impact of an MBI on quality of life among police officers has yet to be scientifically investigated. The POLICE study will also empirically evaluate the influence of MBHP on burnout, anxiety, depression, self-compassion, spirituality, stress, mindfulness, and resilience post-intervention, and at six-month follow-up to assess if potential gains are maintained.

A previous study showed high levels of adherence to a MBI in Brazil [42]. It is relevant to address the feasibility and adherence of this intervention in Brazilian police officers, commonly exposed to dangerous environments and long shifts. We hope to demonstrate, assuming our a priori hypothesis is true, that the health promotion effects of MBIs already demonstrated for a diverse population, is also valid for these high-stress professionals. Great emphasis is placed on physical health cultivation in police training, however evidence-based mental health and quality of life enhancing programs are scarce [82].

One limitation of the study is the use of a waitlist control design, rather than an active control intervention. There is a growing discussion over the use of waitlist in psychological and behavioral research [83]. While there are ethical advantages to a waitlist design because it allows for the provision of care to research participants who are seeking help whilst permitting a non-intervention evaluation, specially in a non-clinical population as ours, it has been noted that such designs may overestimate intervention effects [84].

Despite this limitation, a better understanding of how MBIs may improve police officer mental health is essential to foster the development of future practices, procedures and policies for police institutions around the world. We believe the POLICE study will contribute to this understanding, by seeking to improve physical, emotional and mental fitness, and nourishing the conditions for more effective policing and contributing to society as a whole.

## Abbreviations

DSM-IV: Diagnostic and Statistical Manual of Mental Disorders, 4th Edition
HCPA: Hospital de Clínicas de Porto Alegre - Federal University of Rio Grande do Sul
ICD-10: International Statistical Classification of Diseases and Related Health Problems, 10th Revision
MBHP: Mindfulness-Based Health Promotion
MBI: Mindfulness-Based Intervention
MBIs: Mindfulness-Based Interventions
RCT: Randomized Controlled Trial
UNIFESP: Federal University of São Paulo
